# Toxicological Evaluation and Antimicrobial Activity of a Natural Thymol–Eucalyptol-Based Mixture

**DOI:** 10.3390/toxics13100875

**Published:** 2025-10-14

**Authors:** Boris Lira-Mejía, Luis Barrios-Arpi, Carlos Villaorduña, Tatiana Ancajima, José-Luis Rodríguez, Alejandro Romero, Víctor Puicón, Hugo Patiño

**Affiliations:** 1 GIFATA Research Group, Animal Physiology Laboratory, Faculty of Veterinary Medicine, Major National University of San Marcos, Lima 15021, Peru; 2 Complutox Research Group, Department of Pharmacology and Toxicology, Faculty of Veterinary, Complutense University of Madrid, 28040 Madrid, Spain; manarome@ucm.es; 3 Animal Histopathology Laboratory, Faculty of Veterinary Medicine, National University of San Martín; Tarapoto 22160, Peru; 4 Scientific Area, Feed and Aditive Veterinary Adivet E.I.R.L., Lima 15012, Peru

**Keywords:** thymol–eucalyptol, toxicity, microbiocide activity, chicken, hematology study

## Abstract

Currently, safe alternatives with very low toxicity and good antimicrobial activity are being sought to replace chemical compounds that can be harmful to animal and human health. For this reason, this study evaluated the safety and biofunctional microbiocidal potential of an extract composed of thymol and eucalyptol. Toxicity tests showed low toxicity in both chickens (2000 mg/kg bw) and *Artemia salina* (EC_50_ = 2003 mg/L) and *Daphnia magna* (EC_50_ = 87 mg/L), indicating a safe usage profile. Oxidative stress biomarkers (nitrite and MDA) and antioxidant enzymes (SOD and catalase) improved in treated chickens at 20 days of age. The hematological and biochemical parameters of the treated birds showed normal values similar to those of the control group chickens, with better protein levels and lower AST levels. Histology of the kidney, intestine, and liver showed no changes in any group, confirming the absence of systemic adverse effects. At the molecular level, an improvement in the expression of tight junction proteins (claudin and occludin) was observed, suggesting a strengthening of the intestinal barrier integrity. Finally, the extract demonstrated an antimicrobial effect (*E. coli*, *C. perfringens*, *Salmonella* sp. and *Pseudomonas* sp.) comparable to that of organic acids commonly used as food preservatives, positioning it as a promising alternative in applications.

## 1. Introduction

The increasing global concern regarding food safety together with the evolution of antimicrobial resistance among foodborne pathogens has led to a rapid identification of new alternative preservation methods for the food industry [1,2]. Antimicrobial-resistant foodborne bacteria are of significant concern to public health, whereby over 11% of foodborne pathogens possess antimicrobial resistance, and multidrug-resistant pathogens exceed 36% across all food categories [3]. Pathogens such as E. coli, Salmonella, Pseudomonas, Clostridium perfringens, and species of Aspergillus remain persistent threats to food safety, especially within feed and food production environments [4].

Conventional synthetic antimicrobials, although efficacious, have elicited concerns about their possible adverse effects on human health, including allergic responses, modifications in intestinal microbiota, and facilitation of bacterial resistance development [5,6]. In recent years, consumer demand for natural, clean-label alternatives that comply with food safety requirements and favor less processed products has significantly increased. The United States Food and Drug Administration (FDA) has designated several essential oils as Generally Recognized as Safe (GRAS), such as thyme, turmeric, rosemary, origanum, onion, menthol, among others, establishing a regulatory framework for their application in food systems [7,8].

Essential oils and bioactive agents represent a wide array of potential natural antimicrobials with broad-spectrum activity against bacteria, fungi, and other microorganisms [9]. Thymol and eucalyptol have attracted attention for their antibacterial properties and well-established safety profiles. Thymol, a phenolic monoterpene sourced from Thymus vulgaris and other aromatic flora, exhibits potent antimicrobial properties via multiple pathways, including the disruption of cell membrane integrity, interference with ATP generation, and inhibition of critical cellular functions [10,11]. Research has shown that thymol is effective against a variety of pathogens, with minimum inhibitory concentrations (MICs) ranging from 125 to 250 μg/mL against both Gram-positive and Gram-negative bacteria [12,13]. Eucalyptol (1,8-cineole), the major component of eucalyptus essential oil, has been reported to have potential antimicrobial activity against both bacteria and fungi [14,15]. It acts by altering the permeability of the membrane, which leads to intracellular leakages and morphological changes in microbial cells [16,17]. In this sense, eucalyptol has been well-reported to possess antimicrobial activity against a number of food-related pathogens such as *Staphylococcus aureus*, *E. coli*, and *Candida albicans* [18,19,20].

The antimicrobial activity of some essential oils has been enhanced by combining them with certain compounds such as citric acid, which results in synergistic effects [21,22]. Citric acid not only offers adjuvant antimicrobial action but may also enhance the stability and bioactivity of essential oil components [23]. This approach is also similar to the concept of hurdle technology for the preservation of food, in which combined use of antimicrobial compounds could lead to more effective pathogen control than the use of a single compound [6,24].

However, these natural additives, before being used as commercial agents, need to be fully toxicologically evaluated to determine their safety for the target species as well as for the consumers [25,26]. The chemical variety of the composition of essential oils, which are multicomponent mixtures of toxicologically relevant plant derivatives, requires toxicological investigation using standardized assays [27,28]. Requirements within current models of regulation are to conduct wide ranging toxicological assessments including those for acute and chronic toxicities to derive safe levels of exposure to humans and animals [29].

The development of natural antimicrobial formulations that are applicable for feeds and foods and can satisfy food safety concerns and demands of consumers for natural products is an important milestone [30]. Subsequently, as the safety and efficacy of these formulations remain a concern, they will need to be evaluated through both combined antimicrobial efficacy testing and comprehensive toxicological studies to prevent misuse in the feed and food industry.

Recognizing the significance of natural antimicrobials in maintaining food safety and the need for thorough toxicological assessment of these substances, this study seeks to evaluate the antimicrobial and toxicological profile of a natural blend comprising thymol, eucalyptol, and citric acid. This research will expand the available literature on natural antimicrobials and provide critical safety information needed for regulatory approval and commercial use in the feed and food sector.

## 2. Materials and Methods

### 2.1. Chemicals

The reagents used were of the highest quality and purity. Acetic acid, trichloroacetic acid, thiobarbituric acid, sulfanilamide, *N-1*-napthylethylenediamine dihydrochloride (NED) were used for the TBARS assay and Nitrite levels (Griess assay); target genes and specific primers; microbiology culture media (Sigma-Aldrich, Saint Louis, MO, USA). Natt-Herrick solution was used for blood cell staining (Eurovet, Madrid, Spain). Catalase and SOD kits (Invitrogen, Thermo Fisher Scientific, Waltham, MA, USA) were used for antioxidant assays. RNA extraction Nucleospin RNA plus Kit (Macherey-Nagel, Düren, Germany) was used for RNA extraction. UltraScript^®^ Separate Oligos cDNA Kit (PCRBIOSYSTEMS, London, UK) was used for reverse transcription, and FastGene IC green Universal MIX Master Mix (Nippon Genetics, Düren, Germany) was used for quantitative PCR. Formaldehyde (Fisher Scientific, USA) and propionic acid (Scharlab, Barcelona, España) was used to prepare mix for treatment of corn contaminated with bacteria.

### 2.2. Animals and Experimental Design

This study followed the parameters established by the Bioethics Committee of the Faculty of Veterinary Medicine of the National University of San Marcos (authorization CEBA 2025-17, 16 July 2025) for the use of experimental animals. Authorization was only requested for vertebrates, as according to current regulations, authorization is not required for lower invertebrates such as *Daphnia magna* and *Artemia salina*.

Fourteen male broiler chickens from the Cobb500 line, aged 1 day and 20 days, were used. Feed was administered ad libitum and was appropriate for each stage of growth. Drinking water was administered ad libitum throughout the study period. The bedding consisted of autoclaved wood shavings and was changed weekly to prevent the accumulation of ammonia and microorganisms in the environment. The chickens were raised under standard broiler rearing parameters, with the ambient temperature decreasing weekly according to the requirements of the Cobb500 chicken line.

### 2.3. Toxicity Assay

#### 2.3.1. In Chicken

The natural mixture [NM, eucalyptol (cineole, C_10_H_18_O) and thymol (2-Isopropyl-5-methylphenol, C_10_H_14_O), 1:9, *v*:*v*] with citric acid as carrier, obtained by BIOVET S.A. (Tarragona, Spain) was dissolved in 0.5 mL of distilled water for administration to 1-day-old chickens, while the natural mixture was dissolved in 2 mL of distilled water for oral administration to 20-day-old chickens. In both cases, a single oral dose of 2000 mg/kg body weight of the NM was administered (to 4 chickens aged 1 day, NM1 group; and 4 chickens aged 20 days, NM20 group). Moreover, at each age (1 and 20 days), 3 chickens were used as controls. After oral administration of the NM, the chickens were clinically observed for 14 days after treatment. After this period, the zootechnical parameters were recorded, and the animals were sacrificed by cervical dislocation and subsequent bleeding by sectioning the blood vessels of the neck (jugular vein or carotid artery), and samples of blood, kidney, liver, lung and intestine were taken for subsequent biochemical, histological and molecular analyses (modified from [31,32,33]).

#### 2.3.2. In Model Organisms *Artemia salina*

Young artemia, less than 24 h old, were subjected to acute toxicity testing with the natural mixture. Ten concentrations of the natural mixture (10–1000 mg/L) were used for an exposure time of 48 h. Toxicity records were taken at 24 and 48 h to calculate the LD_50_ at 48 h. Twenty animals were used for each concentration and the control, in triplicate. 9 mL of solution were used for each animal/group. The limit test corresponded to a dose level of 100 mg/L [34].

#### 2.3.3. In Model Organisms *Daphnia magna*

Young daphnia, less than 24 h old, were subjected to acute toxicity testing with the natural mixture. Ten concentrations of the natural mixture (10–10,000 mg/L) were used for an exposure time of 48 h. Toxicity records were taken at 24 and 48 h to calculate the LD_50_ at 48 h. Five animals were used for each concentration and the control, in quadruplicate. 5 mL of solutions were used for each animal/group. The limit test corresponded to a dose level of 100 mg/L [35].

### 2.4. Griess Assay

Blood was collected in EDTA tubes and centrifuged at 3000 rpm/10 min/4 °C. The plasma was separated and kept cold until analysis. 50 µL of plasma was gently mixed with 50 µL of sulfanilamide (dissolved in 5% acetic acid) and incubated for 10 min at room temperature. Then, 50 µL of NED was added and incubated for 10 min at 25 °C [36]. Absorbance was detected at 540 nm (Agilent Technologies, Santa Clara, CA, USA). Previously, proteins were precipitated in plasma samples with 10% trichloroacetic acid.

### 2.5. TBARS Assay

The levels of lipid peroxidation in chicken plasma were evaluated by measuring the levels of malondialdehyde (MDA). Blood samples were collected in tubes containing anticoagulant (EDTA) and centrifuged at 3000 rpm for 10 min. 200 µL of the plasma obtained was mixed with trichloroacetic acid (15%) and centrifuged at 3000 rpm for 10 min. The supernatant was mixed with 400 µL of TBA and incubated at 95 °C for 60 min [37]. After cooling immediately, the absorbance was measured at 532 nm (Agilent Technologies, Santa Clara, CA, USA). Previously, proteins were precipitated in plasma samples with 10% trichloroacetic acid.

### 2.6. Antioxidative Assay

Catalase and SOD activities were determined using a colorimetric assay kit (Invitrogen, Thermo Fisher Scientific, MA, USA) following the manufacturer’s protocol. Plasma was mixed with the specific kit, and this mixture was then used for colorimetric measurement at 560 nm and 450 nm using spectrophotometry (Agilent Technologies, CA, USA).

### 2.7. Biochemical Assay

Specific kits were used for biochemical analysis to assess AST, uric acid, creatinine, alkaline phosphatase, and protein levels (Sigma-Aldrich, USA), according to the manufacturer’s specifications. The reading was performed by spectrophotometry (Agilent Technologies, Santa Clara, CA, USA).

### 2.8. Histopathological Study

For histopathological examination, samples were taken from the liver, intestine, kidney, and lung. All samples were fixed in 10% formalin, processed in paraffin, and stained with conventional hematoxylin and eosin (Supplementary Materials).

### 2.9. Hematology Study

Blood samples were collected during the slaughter of the animals in 3 mL tubes containing ethylenediaminetetraacetic acid (EDTA) anticoagulant, which were immediately homogenized for processing within 20 min of collection. Packed cell volume (PCV) was determined using the microhematocrit method [38], hemoglobin using the cyanmethemoglobin method and read on a spectrophotometer at 540 nm [38,39]. The erythrocyte and leukocyte counts were performed using the Natt and Herrick method, and Natt and Herrick solution for 1:200 dilution were used [40]. For the erythrocyte count, 5 squares of the central grid of the chamber (the 4 corner squares and the central square) were considered, and the resulting sum was multiplied by a factor of 10^3^, expressed in erythrocytes/µL. For the leukocyte count, the number of leukocytes in the 9 grids was counted and 10% of the total obtained was added to the resulting value, multiplying × 200, finally expressing it in leukocytes/µL. MCHC values were obtained from hemoglobin × 100/PCV.

The leukocyte differential count to obtain relative values (%) of heterophils and lymphocytes was determined by microscopic evaluation [41,42]. Heterophil/lymphocyte ratio (H/L ratio, innate and adaptive immune cells) is an indicator of stress on immunity in birds.

### 2.10. Hematology Study

The digestive system was directly exposed to the natural mixture, so 30 mg of small intestine was taken for total RNA extraction using the specifications of the: “Nucleospin RNA plus” extraction kit (Macherey-Nagel, Germany). The purity of the RNA was 1.99 for all samples (260/280 ratio). Complementary DNA (cDNA) was obtained by reverse transcription using the: “UltraScript^®^ Separate Oligos” kit (PCRBIOSYSTEMS, London, United Kingdom). Immediately, 0.1 µg/µL of cDNA was used for quantitative PCR using the specifications of the: “FastGene IC green Universal MIX” Master Mix kit (Nippon Genetics, Germany). The cycle conditions were as follows: initial denaturation at 95 °C for 2 min (1 cycle); denaturation at 95 °C for 5 s (40 cycles); hybridization/elongation at 60–65 °C for 20–30 s (40 cycles). The fold-change gene expression was calculated using the 2^−ΔΔCt^ method, and GAPDH as the normalizing gene [43].

The primer used were listed in Table 1:

### 2.11. Antimicrobial Activity In Vitro

Four bacteria (*E. coli*, *Clostridium perfringens*, *Salmonella* sp., *Pseudomonas aeruginosa*) were used to evaluate the in vitro antimicrobial activity of the natural mixture (NM). In each assay (in triplicate), 100 g of ground yellow corn (sterilized) was used as a substrate, and the corn was contaminated with each pathogenic bacterium at a concentration of 10^6^ CFU g^−1^. The substrates contaminated with bacteria or fungi were treated with NM and a mix of organic acids (F-PA, formaldehyde plus propionic acid) as positive control, at concentrations of 0.5 and 2 L/ton, respectively. The treatments with NM and F-PA were carried out once at a single dose. The plate count of bacteria (*E. coli*, *Clostridium perfringens*, *Salmonella* sp., *Pseudomonas aeruginosa*) was performed 24 h and 7 days post-treatment, in accordance with:

- ISO 4833-2:2013 [44], Microbiology of the food chain — Horizontal method for the enumeration of microorganisms — Part 2: Colony count at 30 °C by the surface plating technique. International Organization for Standardization: Geneva, Switzerland, 2013.
- ISO 16649-1: 2018 [44], Microbiology of the food chain — Horizontal method for the enumeration of beta-glucuronidase-positive Escherichia coli — Part 1: Colony-count technique at 44 °C using membranes and 5-bromo-4-chloro-3-indolyl beta-D-glucuronide. International Organization for Standardization: Geneva, Switzerland, 2018.
- ISO 15213-2:2023 [44], Microbiology of the food chain — Horizontal method for the detection and enumeration of *Clostridium* spp. — Part 2: Enumeration of *Clostridium perfringens* by colony-count technique. International Organization for Standardization: Geneva, Switzerland, 2023.
- ISO 6579-1: 2017 [44], Microbiology of the food chain — Horizontal method for the detection, enumeration and serotyping of Salmonella — Part 1: Detection of Salmonella spp. International Organization for Standardization: Geneva, Switzerland, 2017.
- The results obtained were expressed in bacterial colony-forming units per gram (CFU g^−1^), and as percentages of reduction (%).

### 2.12. Statistical Analysis

The toxicity dose–response curves were analyzed using Origin Pro 8.5.1 (OriginLab, Northampton, MA, USA). Significant differences in the comparative means (mean ± standard deviation) of the in vivo and antimicrobial assays were analyzed using Student’s *t*-test or one-way ANOVA with GraphPad Prism 8.0.1 (GraphPad software, Boston, MA, USA). * *p* < 0.05, ** *p* < 0.01, or *** *p* < 0.001) indicate significant differences.

## 3. Results

### 3.1. Oxidative Stress

**Figure 1:**
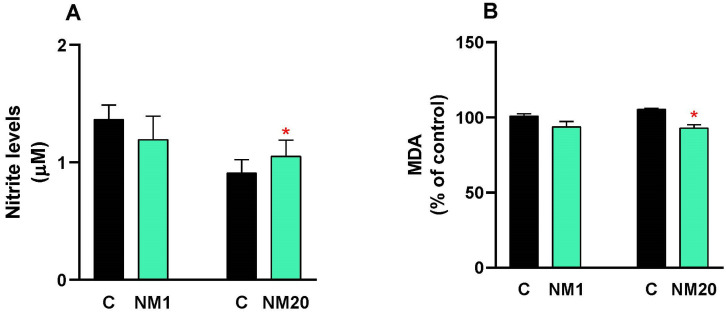
Evaluation of oxidative stress mediators (NO and MDA) in plasma from chickens exposed to a single oral dose of 2000 mg/kg b.w. of a natural mixture of thymol/eucalyptol. NO (**A**) and MDA (**B**) values are expressed as mean ± SD. Statistical differences between the treated groups (NM1 and NM20, gray bar) and the controls (C, black bar) were evaluated using Student’s *t*-test (* *p* ≤ 0.05).

A dose of 2000 mg/kg body weight of the natural mixture of thymol–eucalyptol was administered orally to broiler chickens, and after 14 days, the oxidative stress mediators NO and MDA were analyzed in plasma. NO values in the treated chickens were like the control group (Figure 1A); while only a significant decrease (12%, * *p* ≤ 0.05) in MDA levels was observed in NM20 chickens compared to the control group (Figure 1B).

### 3.2. Antioxidative Assay

**Figure 2:**
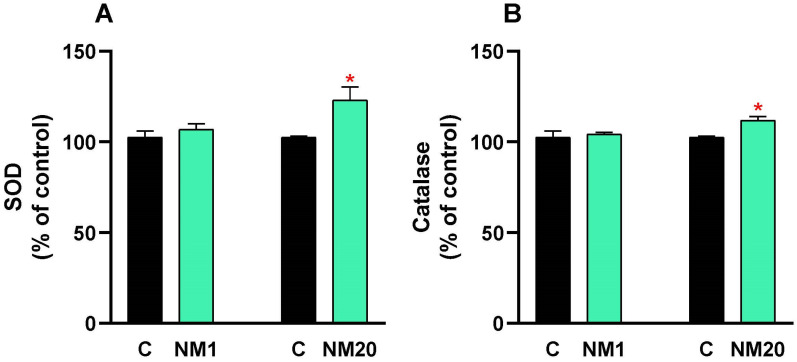
Evaluation of the antioxidant activity (SOD and catalase) in plasma from chickens exposed to a single oral dose of 2000 mg/kg b.w. of a natural mixture of thymol/eucalyptol. SOD (**A**) and catalase (**B**) values are expressed as mean ± SD. Statistical differences between the treated groups (NM1 and NM20, gray bar) and the controls (C, black bar) were evaluated using Student’s *t*-test (* *p* ≤ 0.05).

A dose of 2000 mg/kg body weight of the natural thymol–eucalyptol mixture was administered orally to broiler chickens, and after 14 days, the antioxidant enzymes SOD and catalase were analyzed in plasma. The SOD (Figure 2A) and catalase (Figure 2B) values in NM1 chickens did not change because of exposure to the natural mixture; while in NM20 chickens, there were significant increases in SOD (23%, * *p* ≤ 0.05) and catalase (11%, * *p* ≤ 0.05) activities compared to the control.

### 3.3. Biochemical Assay

**Figure 3:**
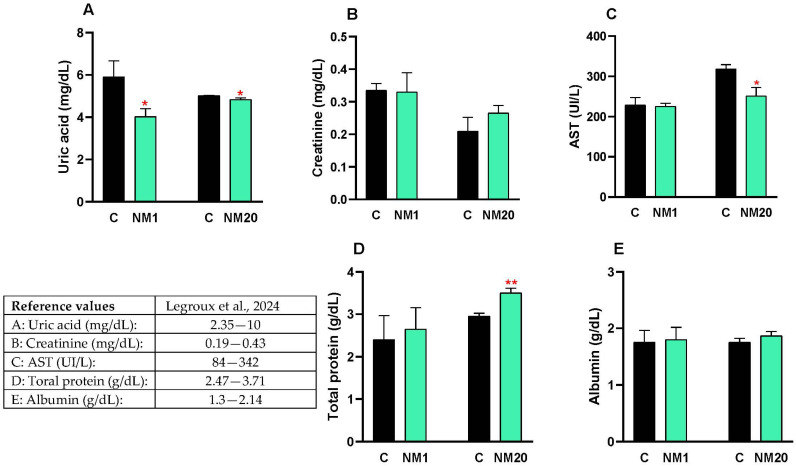
Blood plasma biochemical profile of chickens exposed to a single oral dose of 2000 mg/kg b.w. of a natural mixture of thymol/eucalyptol. The values for uric acid (**A**), creatinine (**B**), AST (**C**), total protein (**D**) and albumin (**E**) are expressed as mean ± SD. Statistical differences between the treated groups (NM1 and NM20, gray bar) and the controls (C, black bar) were evaluated using Student’s *t*-test (* *p* ≤ 0.05; ** *p* ≤ 0.01). The reference values are indicated in the table included in the figure [45].

This study evaluated the blood biochemical profile (uric acid, creatinine, AST, total protein, and albumin) in NM1, NM20, and control chickens. Creatinine (Figure 3B) and albumin (Figure 3E) values did not change in NM1 and NM20 chickens compared to the control group. Uric acid values decreased significantly in the NM1 (22%, * *p* ≤ 0.05) and NM20 (5%, * *p* ≤ 0.05) groups compared to the control (Figure 3A). AST values decreased significantly in the NM20 group (5%, * *p* ≤ 0.05) compared to the control (Figure 3C). Total protein values increased significantly in the NM20 group (19%, ** *p* ≤ 0.01) compared to the control (Figure 3D).

### 3.4. Hematology Study

**Figure 4:**
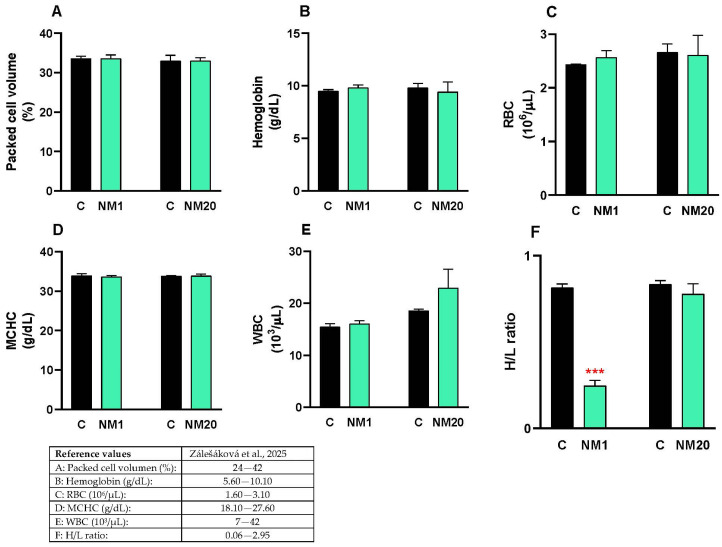
Hematological parameters of chickens administered a single oral dose of 2000 mg/kg body weight of a natural mixture of thymol/eucalyptol. The values for packed cell volume (**A**), hemoglobin (**B**), RBC (**C**), MCHC (**D**), WBC (**E**) and H/L ratio (**F**) are expressed as mean ± SD. Statistical differences between the treated groups (NM1 and NM20, gray bar) and the controls (C, black bar) were evaluated using Student’s *t*-test (*** *p* ≤ 0.001). The reference values are indicated in the table included in the figure [46].

This study evaluated hematological parameters (packed cell volume, hemoglobin, red blood cells, MCHC, white blood cells, and H/L ratio) in NM1, NM20, and control chickens. The values for packed cell volume (Figure 4A), hemoglobin (Figure 4B), red blood cells (Figure 4C), MCHC (Figure 4D), and white blood cells (Figure 4E) did not change in NM1 and NM20 chickens compared to the control group. H/L ratio values decreased significantly in the NM1 groups (70%, *** *p* ≤ 0.001) compared to the control (Figure 4F).

### 3.5. qPCR Assay

**Figure 5:**
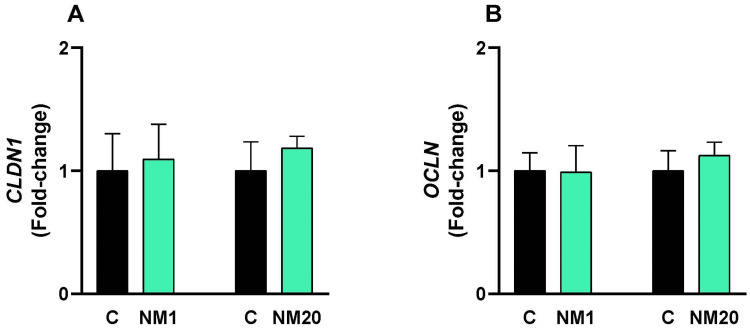
qPCR assay in the intestines of chickens administered a single oral dose of 2000 mg/kg body weight of a natural mixture of thymol/eucalyptol. Fold-change values for claudin (**A**) and occludin (**B**) are expressed as mean ± SD. Statistical differences between the treated groups (NM1 and NM20, gray bar) and the controls (C, black bar) were evaluated using Student’s *t*-test.

A dose of 2000 mg/kg body weight of the natural thymol–eucalyptol mixture was administered orally to broiler chickens, and after 14 days, the molecular expression of the tight junction proteins claudin (Figure 5A) and claudin (Figure 5B) was analyzed in the duodenum. The molecular expression values in the treated chickens were like those in the control group.

### 3.6. Toxicity Study in Artemia salina and Daphnia magna

**Figure 6:**
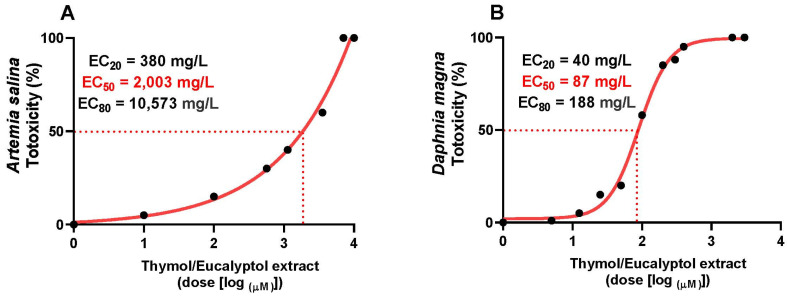
Cytotoxicity induced by thymol/eucalyptol extract (1–10,000 mg/L) on *Artemia salina* (**A**) and *Daphnia magna* (**B**) after 24 h incubation period. Cytotoxicity was determined by viability reduction, and (%) dose–response curve was used to obtain the IC_20,50,80_ values. Data are presented as % of three repetitions.

The dose–response curve showed a characteristic sigmoidal pattern, with an initial phase with no effect at concentrations ≤100 mg/L of the natural mixture, followed by a progressive increase in toxicity between 1000 and 10,000 mg/L of the natural mixture in *Artemia salina*, resulting in an EC_50_ of 2003 mg/L (Figure 6A). On the other hand, the dose–response curve for the toxicity of the natural mixture produced an EC_50_ of 87 mg/mL, indicating a higher sensitivity of *Daphnia magna* to this natural compound (Figure 6B).

### 3.7. Antimicrobial Activity

The microbicidal effect of the natural mixture based on thymol and eucalyptol was evaluated on corn microbiologically contaminated with *E. coli*, *C. perfringens*, *Salmonella* sp. and *Pseudomonas* sp. (106 CFU g^−1^). F-PA was used as a positive control. Table 2 shows that the effect of NM is very similar to that produced by F-PA (positive control) in all the microorganisms analyzed, with a microbial reduction of more than 93% for all bacteria. A better microbicidal effect is also observed at 7 days than at 24 h, both for NM and F-PA.

## 4. Discussion

This study aimed to evaluate the antimicrobial effectiveness and safety characteristics of a naturally sourced compound composed of thymol, eucalyptol, and citric acid. This investigation was motivated by a confluence of issues, including ongoing apprehensions about feed and food safety, the increasing prevalence of resistant strains among foodborne microorganisms, and demonstrated consumer interest in preservation methods using readily recognizable ingredients. The experimental data obtained strongly suggests the safety of use and the potential of this plant-derived mixture as a viable substitute for traditional synthetic antimicrobials in feed and food matrices.

Although synthetic preservatives have traditionally been the mainstay in commercial use, growing anxieties concerning their potential long-term health effects, coupled with increasing regulatory oversight, have necessitated the exploration of naturally occurring substances with proven antimicrobial capabilities by industry participants. The ternary system scrutinized in this study embodies a well-reasoned strategy for leveraging known bioactive elements derived from plant metabolites, specifically targeting the oxidative and membrane-perturbing mechanisms that mediate wide-ranging antimicrobial action.

The natural mixture exhibited impressive antimicrobial activity across a diverse range of foodborne pathogens and fungi, effectively targeting *Escherichia coli*, *Clostridium perfringens*, *Salmonella* sp., *Pseudomonas* sp. When we applied this mixture to microbiologically contaminated yellow corn, we achieved remarkable reductions, over 93% for bacterial populations. These results proved comparable to our positive control (formaldehyde combined with propionic acid). Interestingly, we noticed that antimicrobial effects became more pronounced after seven days compared to the initial 24 h exposure period, suggesting the mixture operates through a sustained, potentially progressive mechanism. This observation is consistent with established research showing that thymol and eucalyptol compromise cell membrane integrity, cause leakage of cellular contents, and disrupt essential metabolic processes [10,11,47]. We believe the citric acid component enhances both the stability and bioactivity of these essential oil constituents [48], which aligns well with hurdle technology principles that combine multiple antimicrobial approaches to boost effectiveness while minimizing the likelihood of microbial adaptation [49,50].

This study demonstrated that NM containing thymol/eucalyptol is safe at high doses (2000 mg/kg b.w.), has an excellent antimicrobial effect, with no reports of antimicrobial resistance, and is safe for animals and humans. However, in our study we used a mixture of organic acids (formaldehyde and propionic acid), and it is currently known that formaldehyde has been widely used in the feed industry as an additive with antimicrobial properties, mainly to reduce bacterial load and control Salmonella in raw materials and balanced feeds [51]. but its handling poses a significant risk to operator health, as chronic exposure by inhalation can cause respiratory tract irritation, dermatitis, and carcinogenic effects [52]. In addition, formaldehyde is a highly corrosive compound that damages metal components in feed production plants, generating additional maintenance costs [53]. In terms of microbiology, although it is effective as a biocide, prolonged use can induce tolerance and resistance mechanisms in certain bacteria, reducing its effectiveness [54]. Finally, its environmental toxicity is also relevant, as studies with model aquatic organisms have shown that formaldehyde is toxic to Daphnia magna (EC50 = 10–17 mg/dL), with acute effects on the survival and development of this invertebrate [35].

However, antimicrobial efficacy alone isn’t sufficient, toxicological safety represents a fundamental requirement for any natural compound intended for food or feed applications [55]. We therefore conducted comprehensive safety evaluations using both in vivo studies and aquatic bioassays. When we administered a single 2000 mg/kg oral dose to broiler chickens, the natural mixture showed a reassuring safety profile across all measured parameters, including hematological, biochemical, and oxidative stress markers [56]. The combination of antimicrobial effectiveness with a well-established safety profile –particularly the absence of detrimental effects on oxidative status and key biochemical parameters– is a critical prerequisite for the practical implementation of plant-derived bioactive compounds in both animal nutrition and strategies for disease management [55,57]. Toxicological evaluations of natural feed additives, including essential oil-based mixtures, show reassuring safety profiles in poultry with no adverse hematological or biochemical effects at recommended doses, aligning with the safety observations described [58,59].

Oxidative stress analysis yielded particularly encouraging results. Nitric oxide levels in treated broiler chickens remained essentially unchanged from controls, while malondialdehyde concentrations—a key indicator of lipid peroxidation—dropped by 12% in 20-day-old chickens. Even more encouraging, we observed enhanced antioxidant defenses: superoxide dismutase activity increased by 23%, and catalase activity rose by 11% in the older chicken group. These findings suggest our natural mixture not only avoids inducing oxidative damage but may provide antioxidant protection in mature broiler chickens [60], potentially boosting their physiological resilience [61]. In this context, studies have shown that improving antioxidant defenses in poultry via natural compounds enhances meat quality and overall health by mitigating damage from ROS and supporting mitochondrial function [62].

The biochemical assessments provided additional confirmation of the safety profile. Markers of creatinine synthesis and liver–kidney function, which play pivotal roles in energy metabolism and tissue development in broilers [63], were consistent with the observed parameters, thereby supporting both safety and metabolic integrity. Furthermore, creatinine and albumin levels remained stable across all age groups, suggesting the absence of detectable renal or hepatic impairment [46,64]. We also noted several beneficial trends: uric acid levels decreased significantly in both 1-day-old (22%) and 20-day-old (5%) chickens, aspartate aminotransferase activity dropped by 5% in older broiler chickens, and total protein levels increased by 19% in the same group. These changes point toward normal or even improved metabolic function rather than any adverse effects [65].

Hematological parameters, including packed cell volume, hemoglobin concentration, and red and white blood cell counts, are widely regarded as reliable markers of overall health and immune status in poultry. Values that fall within normal physiological ranges are typically interpreted as evidence that dietary or management interventions, such as the inclusion of natural feed additives, exert no detrimental effects [66,67]. In our study, hematological profiling revealed no abnormalities: packed cell volume, hemoglobin concentration, red blood cell counts, mean corpuscular hemoglobin concentration, and white blood cell counts all remained within established reference ranges. The heterophil-to-lymphocyte (H/L) ratio, a robust and widely recognized index of physiological stress in chickens, further supported these findings. Notably, a pronounced 70% reduction in the H/L ratio was observed in day-old chicks, a change generally associated with reduced stress responses in chickens [68,69,70]. Equally important to animal health is the maintenance of intestinal barrier integrity, which is mediated by tight junction proteins such as claudin and occludin. Disruption of these proteins by pathogens or toxins can compromise nutrient absorption and overall gut function [71,72]. In this regard, our qPCR analysis of intestinal tissues demonstrated that expression of claudin and occludin remained unchanged, indicating that the natural mixture did not impair tight junction regulation. These results confirm that intestinal barrier function was preserved, a critical prerequisite for maintaining both animal health and nutrient utilization efficiency.

To complement the experimental assays, we performed toxicity assessments using *Artemia salina* and *Daphnia magna*, both of which are well-established model species in aquatic toxicology. These organisms provide rapid and cost-effective bioassays for evaluating the ecotoxicological effects of chemical substances, including essential oils [73,74]. In the present study, *Artemia salina* exhibited an EC_50_ of 2003 mg/L, with no observable effects at concentrations up to 100 mg/L. Conversely, *Daphnia magna* demonstrated markedly higher sensitivity, with an EC_50_ of 87 mg/mL This finding is consistent with previous studies showing that Daphnia magna generally responds more sensitivity to xenobiotics than *Artemia salina*, therefore corroborating the relative susceptibility of *Daphnia magna* to the natural mixture tested [73,75]. Such disparities across species highlight the importance of utilizing diverse species in toxicological assessments when establishing secure exposure limits for intricate ecological networks. This is especially pertinent for essential oil-based compounds, where their chemical diversity and biological effects necessitate thorough investigation to guarantee environmental protection [76,77].

## 5. Conclusions

In conclusion, these results contribute meaningfully to the accumulating evidence supporting natural antimicrobial compounds as viable alternatives for food and feed preservation. The synergistic blend of thymol, eucalyptol, and citric acid demonstrated broad-spectrum antimicrobial efficacy while maintaining acceptable safety profiles across both terrestrial and aquatic bioassays. Such characteristics position this formulation as a compelling plant-derived alternative to conventional synthetic preservatives, aligning with evolving consumer preferences and regulatory frameworks that increasingly favor natural solutions.

## Figures and Tables

**Table 1:** List of primers for tight-junction proteins.

Target Gene	Forward Primer	Reverse Primer
GAPDH	5′-ACTTTGGCATTGTGGAGGGT-3′	5′-GGACGCTGGGATGATGTTCT-3′
Claudin 1 (CLD1)	5′-AGCCTGGCTTAACTGAGTGT-3′	5′-TGCTAGCCGTTGTAGCTGTA-3′
Occludin (OCL)	5′-GTCTGTGGGTTCCTCATC-3′	5′-CCAGTAGATGTTGGCTTTG-3′

**Table 2:** Viable cells count and microbial reduction in feed-maize contaminated with *E. coli*, *C. perfringens*, *Salmonella* sp. and *Pseudomonas* sp. (10^6^ CFU g^−1^) and treated with the microbiocides NM (0.5 L/T) and F-PA (2.0 L/T). The antimicrobial analysis was performed 24 h and 7 days after treatment with NM or F-PA.

	Microbiocide	Time Exposure					
		24 h			7 Days		
		CFU g^−1^	S.D.	MR (%)	CFU g^−1^	S.D.	MR (%)
***E. coli***	BN (0.5 L/T)	6.5 × 10^4^ *a	1.0 × 10^3^	96	3.3 × 10^4^ *b	2.2 × 10^3^	98
	F-PA (2 L/T)	2.7 × 10^4^ *a	0.7 × 10^3^	98	0.6 × 10^4^ *b	0.2 × 10^3^	99
	Control (-)	1.5 × 10^6^ **a**	5.3 × 10^4^	-	1.7 × 10^6^ **a**	2.5 × 10^4^	-

***C. perfringens***	BN (0.5 L/T)	8.31 × 10^4^ *a	2.5 × 10^3^	93	1.3 × 10^4^ *b	1.4 × 10^3^	99
	F-PA (2 L/T)	5.7 × 10^4^ *a	2.5 × 10^3^	95	0.7 × 10^4^ *b	0.1 × 10^3^	99
	Control (-)	1.2 × 10^6^ **a**	3.6 × 10^4^	-	1.2 × 10^6^ **a**	2.5 × 10^4^	-

***Salmonella* sp.**	BN (0.5 L/T)	7.3 × 10^4^ *a	1.8 × 10^3^	94	2.6 × 10^4^ *b	0.4 × 10^3^	98
	F-PA (2 L/T)	2.4 × 10^4^ *a	0.3 × 10^3^	97	0.1 × 10^4^ *b	1.0 × 10^3^	99
	Control (-)	1.1 × 10^6^ **a**	5.0 × 10^4^	-	1.2 × 10^6^ **a**	4.1 × 10^4^	-

***P. aeruginosa***	BN (0.5 L/T)	6.2 × 10^4^ *a	2.5 × 10^3^	95	4.1 × 10^4^ *b	1.5 × 10^3^	97
	F-PA (2 L/T)	3.0 × 10^4^ *a	2.5 × 10^3^	97	1.4 × 10^4^ *b	0.3 × 10^3^	99
	Control (-)	1.1 × 10^6^ **a**	2.0 × 10^4^	-	1.2 × 10^6^ **a**	4.1 × 10^4^	-

* Indicates significant differences (*p* < 0.05) between treatment groups and control group. ^**a**,**b**^ Significant difference (*p* < 0.05) between expo-sure times (24 h vs. 7 days).

## Data Availability

The original contributions presented in this study are included in the article/supplementary material. Further inquiries can be directed to the corresponding author.

## Supplementary Materials

The following supporting information can be downloaded at: https://www.mdpi.com/article/10.3390/toxics13100875/s1, Figure S1: Sections of renal histology (A, control; B, NM), small intestine (C, control; D, NM) and liver (E, control; F, NM) from 15-day-old chickens that received a dose of 2000 mg/kg b.w. of a natural mixture (NM) of thymol/eucalyptol. Kidney: apparently normal histological patterns are observed in both groups, with clearly defined tubules and glomeruli whose mesangial cells are differentiated within the space delimited by Bowman’s capsule. Liver: preserved hepatic trabeculae with clear delimitation of the hepatic sinusoids surrounding the centrilobular vein in both the control and treated groups. Intestine: the treated group showed no structural changes compared to the control group; differentiation of the intestinal crypts at the base of the intestinal mucosa was observed and clear visualisation of the submucosa was noted; Figure S2: Sections of renal histology (A, control; B, NM), small intestine (C, control; D, NM) and liver (E, control; F, NM) from 34-day-old chickens that received a dose of 2000 mg/kgb.w. of a natural mixture (NM) of thymol/eucalyptol. Both groups showed similar histological characteristics, indicating that the dose of 2000 mg/kg body weight did not cause cytostructural alterations in the tissues studied. Kidney: the renal tubules are clearly defined, with distinctive characteristic nuclei and intact renal glomeruli, and a small number of red blood cells in the interstitial blood vessels. In the intestine, the mucosa shows the base of the intestinal villi with clearly defined crypts. In the liver, the preserved tissue architecture shows clearly defined hepatic cords and sinusoidal capillaries with an incipient amount of red blood cells.

## References

[1] Wu-Wu J.W.F., Guadamuz-Mayorga C., Oviedo-Cerdas D., Zamora W.J. (2023). Antibiotic Resistance and Food Safety: Perspectives on New Technologies and Molecules for Microbial Control in the Food Industry. Antibiotics.

[2] Hashempour-Baltork F., Hosseini H., Shojaee-Aliabadi S., Torbati M., Alizadeh A.M., Alizadeh M. (2019). Drug Resistance and the Prevention Strategies in Food Borne Bacteria: An Update Review. Adv. Pharm. Bull..

[3] Tao Q., Wu Q., Zhang Z., Liu J., Tian C., Huang Z., Malakar P.K., Pan Y., Zhao Y. (2022). Meta-Analysis for the Global Prevalence of Foodborne Pathogens Exhibiting Antibiotic Resistance and Biofilm Formation. Front. Microbiol..

[4] Karanth S., Feng S., Patra D., Pradhan A.K. (2023). Linking Microbial Contamination to Food Spoilage and Food Waste: The Role of Smart Packaging, Spoilage Risk Assessments, and Date Labeling. Front. Microbiol..

[5] Farid N., Waheed A., Motwani S. (2023). Synthetic and Natural Antimicrobials as Control against Food Borne Pathogens: A Review. Heliyon.

[6] Teshome E., Forsido S.F., Rupasinghe H.P.V., Olika Keyata E. (2022). Potentials of Natural Preservatives to Enhance Food Safety and Shelf Life: A Review. Sci. World J..

[7] Hyldgaard M., Mygind T., Meyer R.L. (2012). Essential Oils in Food Preservation: Mode of Action, Synergies, and Interactions with Food Matrix Components. Front. Microbiol..

[8] Angane M., Swift S., Huang K., Butts C.A., Quek S.Y. (2022). Essential Oils and Their Major Components: An Updated Review on Antimicrobial Activities, Mechanism of Action and Their Potential Application in the Food Industry. Foods.

[9] Maurya A., Prasad J., Das S., Dwivedy A.K. (2021). Essential Oils and Their Application in Food Safety. Front. Sustain. Food Syst..

[10] Nagoor Meeran M.F., Javed H., Al Taee H., Azimullah S., Ojha S.K. (2017). Pharmacological Properties and Molecular Mechanisms of Thymol: Prospects for Its Therapeutic Potential and Pharmaceutical Development. Front. Pharmacol..

[11] Chouhan S., Sharma K., Guleria S. (2017). Antimicrobial Activity of Some Essential Oils—Present Status and Future Perspectives. Medicines.

[12] Gan C., Langa E., Valenzuela A., Ballestero D., Pino-Otín M.R. (2023). Synergistic Activity of Thymol with Commercial Antibiotics against Critical and High WHO Priority Pathogenic Bacteria. Plants.

[13] Khwaza V., Aderibigbe B.A. (2025). Antibacterial Activity of Selected Essential Oil Components and Their Derivatives: A Review. Antibiotics.

[14] Aleksic Sabo V., Knezevic P. (2019). Antimicrobial Activity of *Eucalyptus camaldulensis* Dehn. Plant Extracts and Essential Oils: A Review. Ind. Crop. Prod..

[15] Salvatori E.S., Morgan L.V., Ferrarini S., Zilli G.A.L., Rosina A., Almeida M.O.P., Hackbart H.C.S., Rezende R.S., Albeny-Simões D., Oliveira J.V. (2023). Anti-Inflammatory and Antimicrobial Effects of *Eucalyptus* spp. Essential Oils: A Potential Valuable Use for an Industry Byproduct. Evid. Based Complement. Alternat. Med..

[16] Hoch C.C., Petry J., Griesbaum L., Weiser T., Werner K., Ploch M., Verschoor A., Multhoff G., Bashiri Dezfouli A., Wollenberg B. (2023). 1,8-Cineole (Eucalyptol): A Versatile Phytochemical with Therapeutic Applications across Multiple Diseases. Biomed. Pharmacother..

[17] Zengin H., Baysal A.H. (2014). Antibacterial and Antioxidant Activity of Essential Oil Terpenes against Pathogenic and Spoilage-Forming Bacteria and Cell Structure-Activity Relationships Evaluated by SEM Microscopy. Molecules.

[18] Mączka W., Duda-Madej A., Górny A., Grabarczyk M., Wińska K. (2021). Can Eucalyptol Replace Antibiotics?. Molecules.

[19] Merghni A., Belmamoun A.R., Urcan A.C., Bobiş O., Lassoued M.A. (2023). 1,8-Cineol (Eucalyptol) Disrupts Membrane Integrity and Induces Oxidative Stress in Methicillin-Resistant *Staphylococcus aureus*. Antioxidant.

[20] Gupta P., Pruthi V., Poluri K.M. (2021). Mechanistic Insights into *Candida* Biofilm Eradication Potential of Eucalyptol. J. Appl. Microbiol..

[21] Li X.S., Xue J.Z., Qi Y., Muhammad I., Wang H., Li X.Y., Luo Y.J., Zhu D.M., Gao Y.H., Kong L.C. (2023). Citric Acid Confers Broad Antibiotic Tolerance through Alteration of Bacterial Metabolism and Oxidative Stress. Int. J. Mol. Sci..

[22] Su L.C., Xie Z., Zhang Y., Nguyen K.T., Yang J. (2014). Study on the Antimicrobial Properties of Citrate-Based Biodegradable Polymers. Front. Bioeng. Biotechnol..

[23] Burel C., Kala A., Purevdorj-Gage L. (2021). Impact of pH on Citric Acid Antimicrobial Activity against Gram-Negative Bacteria. Lett. Appl. Microbiol..

[24] Karnwal A., Malik T. (2024). Exploring the Untapped Potential of Naturally Occurring Antimicrobial Compounds: Novel Advancements in Food Preservation for Enhanced Safety and Sustainability. Front. Sustain. Food Syst..

[25] Pinto L., Tapia-Rodríguez M.R., Baruzzi F., Ayala-Zavala J.F. (2023). Plant Antimicrobials for Food Quality and Safety: Recent Views and Future Challenges. Foods.

[26] Li S., Jiang S., Jia W., Guo T., Wang F., Li J., Yao Z. (2024). Natural Antimicrobials from Plants: Recent Advances and Future Prospects. Food Chem..

[27] Stevanović Z.D., Bošnjak-Neumüller J., Pajić-Lijaković I., Raj J., Vasiljević M. (2018). Essential Oils as Feed Additives—Future Perspectives. Molecules.

[28] Sartori Tamburlin I., Roux E., Feuillée M., Labbé J., Aussaguès Y., El Fadle F.E., Fraboul F., Bouvier G. (2021). Toxicological Safety Assessment of Essential Oils Used as Food Supplements to Establish Safe Oral Recommended Doses. Food Chem. Toxicol..

[29] Xie K., Tashkin D.P., Luo M.Z., Zhang J.Y. (2019). Chronic Toxicity of Inhaled Thymol in Lungs and Respiratory Tracts in Mouse Model. Pharmacol. Res. Perspect..

[30] Negi P.S. (2012). Plant Extracts for the Control of Bacterial Growth: Efficacy, Stability and Safety Issues for Food Application. Int. J. Food Microbiol..

[31] Sani D., Abdu P.A., Mamman M., Jolayemi K.O., Yusuf P.O., Andamin A.D. (2021). Research Note: Evaluation of Acute Oral Toxicity of Povidone-Iodine in Cockerels Using the Up-and-Down Procedure. Poult. Sci..

[32] OECD (1984). Test No. 205: Avian Dietary Toxicity Test. OECD Guidelines for the Testing of Chemicals, Section 2.

[33] OECD (2016). Test No. 223: Avian Acute Oral Toxicity Test. OECD Guidelines for the Testing of Chemicals, Section 2.

[34] Nunes B.S., Carvalho F.D., Guilhermino L.M., Van Stappen G. (2006). Use of the Genus *Artemia* in Ecotoxicity Testing. Environ. Pollut..

[35] OECD (2004). Test No. 202: *Daphnia* sp. Acute Immobilisation Test. OECD Guidelines for the Testing of Chemicals, Section 2.

[36] Green L.C., Wagner D.A., Glogowski J., Skipper P.L., Wishnok J.S., Tannenbaum S.R. (1982). Analysis of Nitrate, Nitrite, and [15N]Nitrate in Biological Fluids. Anal. Biochem..

[37] Placer Z.A., Cushman L.L., Johnson B.C. (1966). Estimation of Product of Lipid Peroxidation (Malonyl Dialdehyde) in Biochemical Systems. Anal. Biochem..

[38] Campbell T.W. (2015). Exotic Animal Hematology and Cytology.

[39] Clark P., Boardman W., Raidal S. (2009). Atlas of Clinical Avian Hematology.

[40] Natt M.P., Herrick C.H.A. (1952). A New Blood Diluent for Counting the Erythrocytes and Leucocytes of the Chicken. Poult. Sci..

[41] Lobato E., Moreno J., Merino S., Sanz J.J., Arriero E. (2005). Haematological Variables Are Good Predictors of Recruitment in Nestling Pied Flycatchers (*Ficedula hypoleuca*). Écoscience.

[42] Masello J.F., Choconi R.G., Helmer M., Kremberg T., Lubjuhn T., Quillfeldt P. (2009). Do Leucocytes Reflect Condition in Nestling Burrowing Parrots *Cyanoliseus patagonus* in the Wild?. Comp. Biochem. Physiol. A.

[43] Lira-Mejía B., Calderon-Romero R., Ordaya-Fierro J., Medina C., Rodríguez J.L., Romero A., Dávila R., Ramos-Gonzalez M. (2025). Impact of Exposure Duration to High-Altitude Hypoxia on Oxidative Homeostasis in Rat Brain Regions. Int. J. Mol. Sci..

[44] ISO (2025). Microbiology of the Food Chain—Horizontal Method.

[45] Legroux D., Kersten L., Barral G., Mauras A., Buronfosse T., Ramery E. (2025). Evaluation of blood erythroid parameters in male broiler chickens (Ross 308) with the Sysmex XT-2000iV and Sysmex XN-1000V analyzers and determination of hematological reference intervals obtained with manual and instrumental methods. Vet. Clin. Pathol..

[46] Zálešáková D., Novotný J., Řiháček M., Horáková L., Mrkvicová E., Šťastník O., Pavlata L. (2025). The blood biochemical parameters intervals and dynamics in modern broiler chickens. Vet. Anim. Sci..

[47] Batista D.G., Sganzerla W.G., da Silva L.R., Vieira Y.G.S., Almeida A.R., Dominguini D., Ceretta L., Pinheiro A.C., Bertoldi F.C., Becker D. (2024). Antimicrobial and Cytotoxic Potential of Eucalyptus Essential Oil-Based Nanoemulsions for Mouthwashes Application. Antibiotics.

[48] Park J.H., Kim S., Chang Y., Imm J.Y. (2022). Synergistic antimicrobial effect and mode of action of palmarosa oil-loaded nanoemulsion and citric acid against *Pectobacterium carotovorum*. Food Sci. Biotechnol..

[49] Ghahari A., Khosravi-Darani K. (2024). Hurdle technology using enzymes and essential oil to remove biofilm and increase the effectiveness of this process with the microencapsulation method. Food Sci. Nutr..

[50] Mechmechani S., Khelissa S., Gharsallaoui A., Omari K.E., Hamze M., Chihib N.E. (2022). Hurdle technology using encapsulated enzymes and essential oils to fight bacterial biofilms. Appl. Microbiol. Biotechnol..

[51] EFSA Panel on Additives and Products or Substances Used in Animal Feed (FEEDAP) (2014). Scientific Opinion on the Safety and Efficacy of Formaldehyde as a Feed Hygiene Substance in Feed for Pigs and Poultry. EFSA J..

[52] Sun X., Yang C., Zhang W., Zheng J., Ou J., Ou S. (2025). Toxicity of Formaldehyde, and Its Role in the Formation of Harmful and Aromatic Compounds during Food Processing. Food Chem. X.

[53] Faria D.L., Cavicchioli A., Puglieri T.S. (2010). Indoors Lead Corrosion: Reassessing the Role of Formaldehyde. Vib. Spectrosc..

[54] Wollmann A., Kaulfers P.M. (1991). Formaldehyde-Resistance in *Enterobacteriaceae* and *Pseudomonas aeruginosa*: Identification of Resistance Genes by DNA-Hybridization. Zentralblatt Hyg. Umweltmed. Int. J. Hyg. Environ. Med..

[55] Vilas-Boas A.A., Pintado M., Oliveira A.L.S. (2021). Natural Bioactive Compounds from Food Waste: Toxicity and Safety Concerns. Foods.

[56] Surai P.F., Kochish I.I., Fisinin V.I., Kidd M.T. (2019). Antioxidant Defence Systems and Oxidative Stress in Poultry Biology: An Update. Antioxidants.

[57] Coudert E., Baéza E., Chartrin P., Jimenez J., Cailleau-Audouin E., Bordeau T., Berri C. (2023). Slow and Fast-Growing Chickens Use Different Antioxidant Pathways to Maintain Their Redox Balance during Postnatal Growth. Animals.

[58] Srinivasa Rao B., Chandrasekaran C.V., Srikanth H.S., Sasikumar M., Edwin Jothie R., Haseena B., Bharathi B., Selvam R., Prashanth D. (2018). Mutagenicity and Acute Oral Toxicity Test for Herbal Poultry Feed Supplements. J. Toxicol..

[59] Bampidis V., Azimonti G., Bastos M.L., Christensen H., Dusemund B., Durjava M., Kouba M., López-Alonso M., López Puente S., EFSA Panel on Additives and Products or Substances used in Animal Feed (FEEDAP) (2023). Safety and efficacy of a feed additive consisting of a preparation of essential oils of thyme and star anise, and quillaja bark powder (BIOSTRONG^®^ 510 all natural) for all poultry species (Delacon Biotechnik GmbH). EFSA J..

[60] Sierżant K., Piksa E., Konkol D., Lewandowska K., Asghar M.U. (2023). Performance and antioxidant traits of broiler chickens fed with diets containing rapeseed or flaxseed oil and optimized quercetin. Sci. Rep..

[61] Kouvedaki I., Pappas A.C., Surai P.F., Zoidis E. (2024). Nutrigenomics of Natural Antioxidants in Broilers. Antioxidants.

[62] Chen Z., Xing T., Li J., Zhang L., Jiang Y., Gao F. (2022). Oxidative stress impairs the meat quality of broiler by damaging mitochondrial function, affecting calcium metabolism and leading to ferroptosis. Anim. Biosci..

[63] Dayan J., Melkman-Zehavi T., Reicher N., Braun U., Inhuber V., Mabjeesh S.J., Halevy O., Uni Z. (2023). Supply and demand of creatine and glycogen in broiler chicken embryos. Front. Physiol..

[64] Abo Ghanima M.M., Abd El-Hack M.E., Al-Otaibi A.M., Nasr S., Almohmadi N.H., Taha A.E., Jaremko M., El-Kasrawy N.I. (2023). Growth performance, liver and kidney functions, blood hormonal profile, and economic efficiency of broilers fed different levels of threonine supplementation during feed restriction. Poult. Sci..

[65] Tesser G.L.S., Avila A.S., Broch J., Souza C., Polese C., Kaufmann C., Eyng C., Savaris V.D.L., Junior N.R., Bruno L.D.G. (2022). Performance, metabolism, and meat quality of broilers fed dry brewery residue. Trop. Anim. Health Prod..

[66] Dalia A.M., Loh T.C., Sazili A.Q., Samsudin A.A. (2020). Influence of bacterial organic selenium on blood parameters, immune response, selenium retention and intestinal morphology of broiler chickens. BMC Vet. Res..

[67] Righi F., Pitino R., Manuelian C.L., Simoni M., Quarantelli A., De Marchi M., Tsiplakou E. (2021). Plant Feed Additives as Natural Alternatives to the Use of Synthetic Antioxidant Vitamins on Poultry Performances, Health, and Oxidative Status: A Review of the Literature in the Last 20 Years. Antioxidants.

[68] Gross W.B., Siegel H.S. (1983). Evaluation of the heterophil/lymphocyte ratio as a measure of stress in chickens. Avian Dis..

[69] Al-Murrani W.K., Kassab A., al-Sam H.Z., al-Athari A.M. (1997). Heterophil/lymphocyte ratio as a selection criterion for heat resistance in domestic fowls. Br. Poult. Sci..

[70] Gil M.G., Gomez-Raya L., Torres O., Cigarroa-Vazquez F.A., Davila S.G., Rauw W.M. (2023). Heterophil/lymphocyte response of local Spanish breeds of laying hens to cold stress, heat stress, and water restriction. J. Therm. Biol..

[71] von Buchholz J.S., Ruhnau D., Hess C., Aschenbach J.R., Hess M., Awad W.A. (2022). Paracellular intestinal permeability of chickens induced by DON and/or *C. jejuni* is associated with alterations in tight junction mRNA expression. Microb. Pathog..

[72] Awad W.A., Hess C., Hess M. (2017). Enteric Pathogens and Their Toxin-Induced Disruption of the Intestinal Barrier through Alteration of Tight Junctions in Chickens. Toxins.

[73] Macrì A., Stazi A.V., Di Delupis G.D. (1988). Acute toxicity of furazolidone on *Artemia salina*, *Daphnia magna*, and *Culex pipiens molestus* larvae. Ecotoxicol. Environ. Saf..

[74] Favilla M., Macchia L., Gallo A., Altomare C. (2006). Toxicity assessment of metabolites of fungal biocontrol agents using two different (*Artemia salina* and *Daphnia magna*) invertebrate bioassays. Food Chem. Toxicol..

[75] Honda M., Suzuki N. (2020). Toxicities of Polycyclic Aromatic Hydrocarbons for Aquatic Animals. Int. J. Environ. Res. Public Health.

[76] Cavion F., Pelin M., Ponti C., Della Loggia R., Tubaro A., Sosa S. (2022). Ecotoxicological Impact of the Marine Toxin Palytoxin on the Micro-Crustacean *Artemia franciscana*. Mar. Drugs.

[77] Salmani M.H., Garzegar S., Ehrampoush M.H., Askarishahi M. (2021). Predicting anionic surfactant toxicity to *Daphnia magna* in aquatic environment: A green approach for evaluation of EC50 values. Environ. Sci. Pollut. Res. Int..

