# Supplementary material for: Toxicological Evaluation and Antimicrobial Activity of a Natural Thymol–Eucalyptol-Based Mixture
Source: Toxics. 2025 Oct 14;13(10):875. doi: 10.3390/toxics13100875 (PMC12568283; doi:10.3390/toxics13100875)
Supplement: Supplementary file 1 [file toxics-13-00875-s001.zip › toxics-3894411-supplementary.pdf]

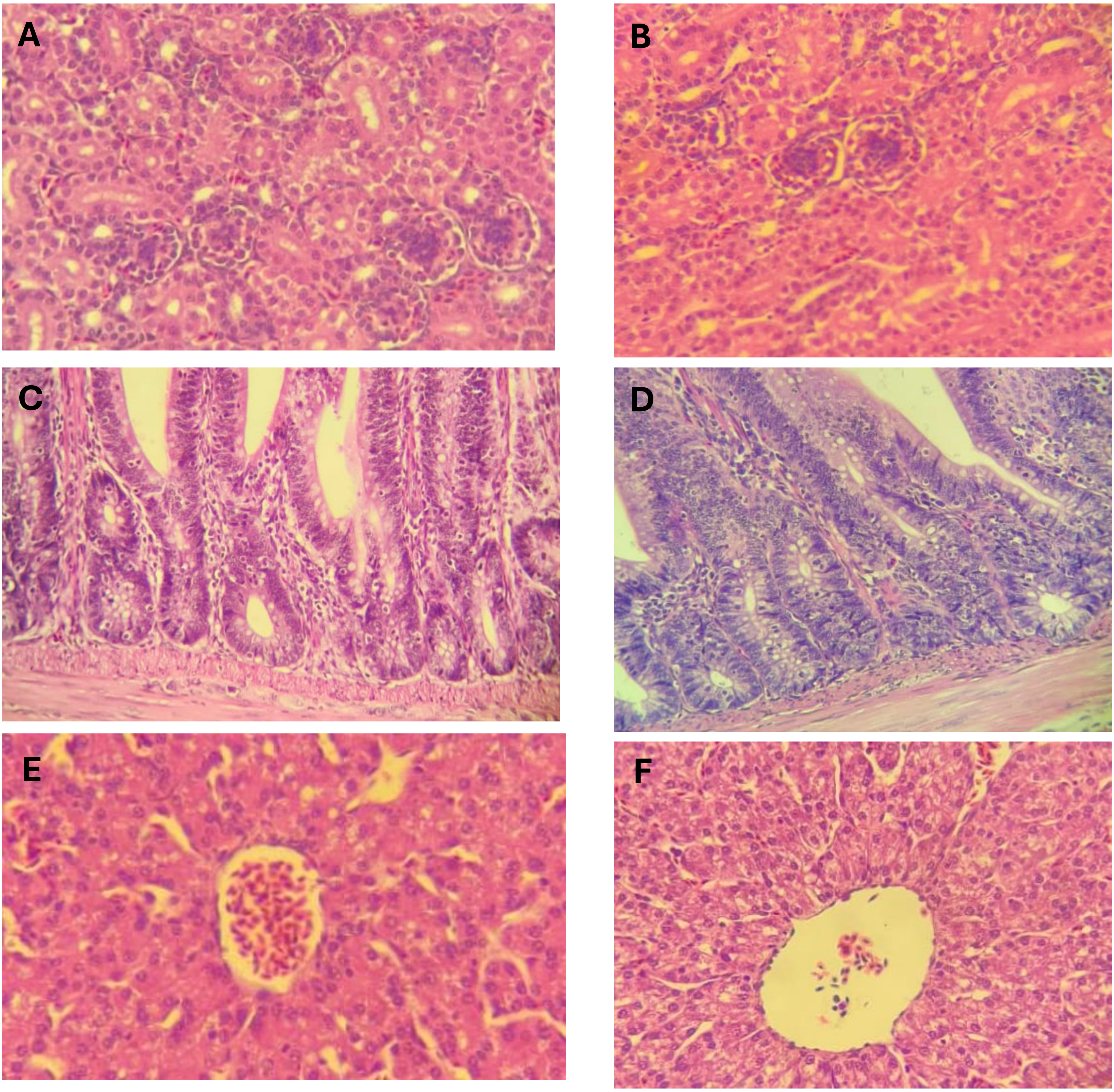

Figure S1. Sections of renal histology (A, control; B, NM), small intestine (C, control; D, NM) and liver (E, control; F, NM) from 15-day-old chickens that received a dose of 2000 mg/kg b.w. of a natural mixture (NM) of thymol/eucalyptol. Kidney: apparently normal histological patterns are observed in both groups, with clearly defined tubules and glomeruli whose mesangial cells are differentiated within the space delimited by Bowman's capsule. Liver: preserved hepatic trabeculae with clear delimitation of the hepatic sinusoids surrounding the centrilobular vein in both the control and treated groups. Intestine: the treated group showed no structural changes compared to the control group; differentiation of the intestinal crypts at the base of the intestinal mucosa was observed and clear visualisation of the submucosa was noted.

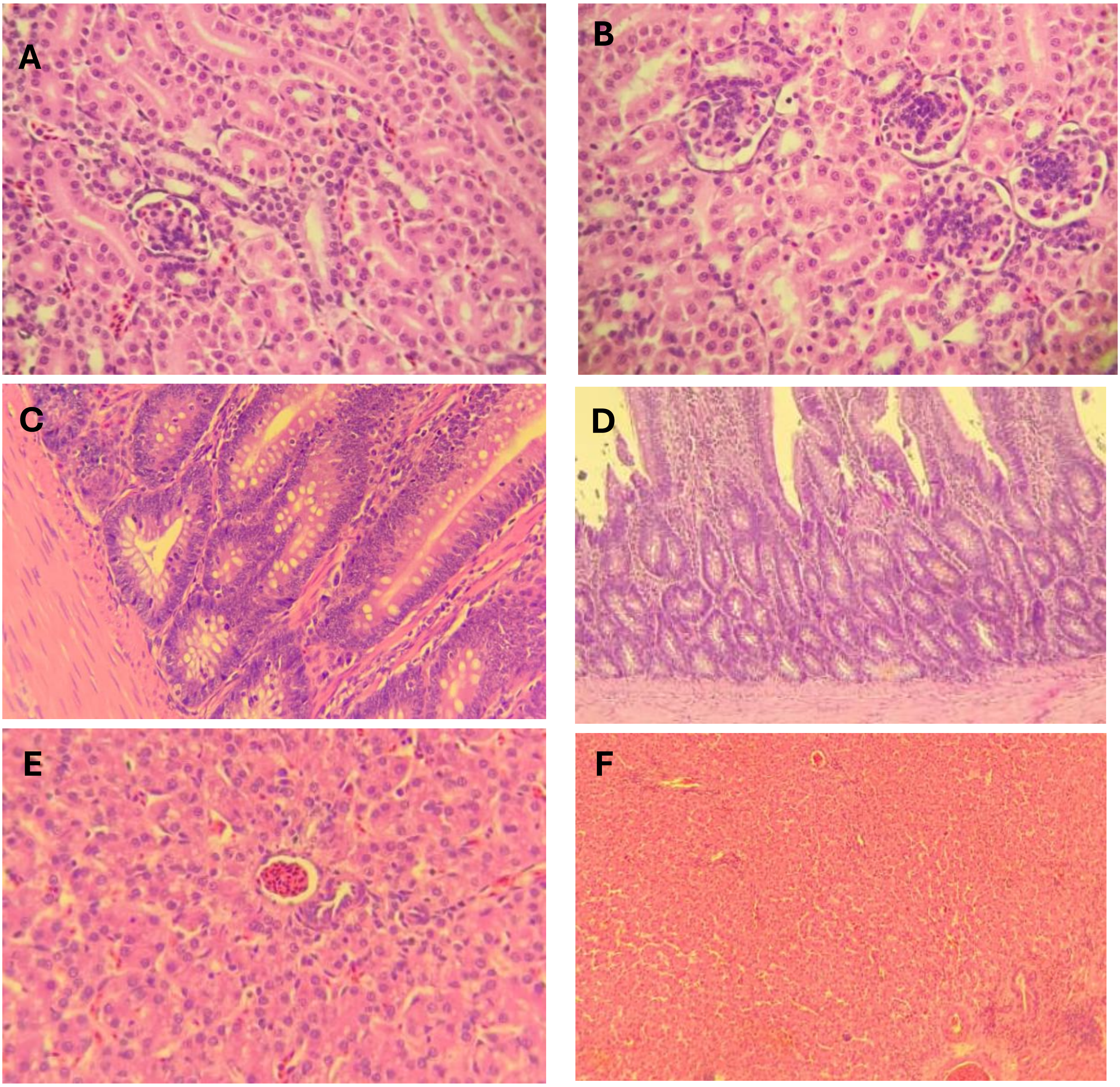

Figure S2. Sections of renal histology (A, control; B, NM), small intestine (C, control; D, NM) and liver (E, control; F, NM) from 34-day-old chickens that received a dose of 2000 mg/kgb.w. of a natural mixture (NM) of thymol/eucalyptol. Both groups showed similar histological characteristics, indicating that the dose of 2000 mg/kg body weight did not cause cytostructural alterations in the tissues studied. Kidney: the renal tubules are clearly defined, with distinctive characteristic nuclei and intact renal glomeruli, and a small number of red blood cells in the interstitial blood vessels. In the intestine, the mucosa shows the base of the intestinal villi with clearly defined crypts. In the liver, the preserved tissue architecture shows clearly defined hepatic cords and sinusoidal capillaries with an incipient amount of red blood cells.
